# Supplementary material for: Experimental infection of Mexican free-tailed bats (Tadarida brasiliensis) with SARS-CoV-2
Source: bioRxiv. 2022 Jul 19:2022.07.18.500430. Preprint. [Version 2] doi: 10.1101/2022.07.18.500430 (PMC9327625; doi:10.1101/2022.07.18.500430)
Supplement: 1 [file NIHPP2022.07.18.500430V2-supplement-1.pdf]

bioRxiv preprint doi: https://doi.org/10.1101/2022.07.18.500430; this version posted July 19, 2022. The copyright holder for this preprint (which was not certified by peer review) is the author/funder. This article is a US Government work. It is not subject to copyright under 17 USC 105 and is also made available for use under a CC0 license.

Supplemental Table 1. Rabies diagnostic results from Mexican free-tailed bats by direct fluorescent antibody (DFA) and the presence of alpha- or betacoronaviruses prior to initiation of the SARS-CoV-2 challenge study.

Bat ID	Age	Treatment	DFA[2]	Coronavirus
101	j	None[1]	Pos	Neg
105	j	None[1]	Neg	Neg
107	j	None[1]	Neg	Neg
115	j	None[1]	Neg	Neg
119	j	None[1]	Neg	Neg
102	j	Control	Neg	Neg
108	j	Control	Neg	Neg
104	a	Inoculated	Neg	Neg
103	j	Transmission	Neg	Neg
112	j	Inoculated	Neg	Neg
113	a	Transmission	Neg	Neg
110	a	Inoculated	Neg	Neg
117	a	Transmission	Neg	Neg
118	a	Inoculated	Neg	Neg
114	j	Transmission	Neg	Neg
120	j	Inoculated	Neg	Neg
121	j	Transmission	Neg	Neg
124	j	Inoculated	Neg	Neg
125	a	Transmission	Neg	Neg
111	j	Inoculated	Neg	Neg
109	j	Transmission	Neg	Neg
123	a	Inoculated	Neg	Neg
122	j	Transmission	Neg	Neg
127	a	Inoculated	Neg	Neg
128	a	Transmission	Neg	Neg
129	j	Inoculated	Neg	Neg

[1]None - Bat was euthanized prior to SARS-CoV-2 challenge
[2]Pos – rabies virus presence in brain tissue; Neg – rabies virus abscent in brain tissue or coronavirus abscent in fecal material

bioRxiv preprint doi: https://doi.org/10.1101/2022.07.18.500430; this version posted July 19, 2022. The copyright holder for this preprint (which was not certified by peer review) is the author/funder. This article is a US Government work. It is not subject to copyright under 17 USC 105 and is also made available for use under a CC0 license.

Supplemental Table 2

Variant detection in SARS-CoV-2 isolated from Bat 118, DPI 8. Genetic changes in the SARS-CoV-2 inoculum strain (USA-WA1/2020; Genbank MN985325) and Bat 118 DPI 8 (Genbank OM995890) after one passage in Vero cells compared with the reference strain (Wuhan HU-1; Genbank MN908947). Sample ID- sample name. Reference position- location of genetic variant on Wuhan HU-1 genome. Reference sequence- nucleotide sequence in HU-1. Variant- nucleotide in WA-1 and bat isolate. Count- number of reads covering the variant. Frequency- proportions of reads with variant. AA change- change in amino acid coding affected by variant. Syn.- synonymous

**Sample ID**	**Reference Position**	**Reference Sequence**	**Variant**	**Count/frequency**	**AA Change**
WA-1	8782	C	T	3628/98.45	Syn.
	28144	T	C	282/96.58	Syn.
Bat 118 DPI 8	8782	C	T	3514/98.46	Syn.
	28144	T	C	311/97.49	Syn.
	28253	C	T	1509/94.85	Syn.
	28603	C	T	3928/90.05	Syn.

Supplemental Table 3. SARS-CoV-2 antigen distribution in selected tissues from SARS-CoV-2 inoculated and uninoculated control Mexican free-tailed bats. Bats euthanized, necropsied and tissue samples collected on day post-inoculation (DPI) 7 (bats 127, 128)); DPI 10 (bat 112); DPI 14 (bats 103, 104); and DPI 20 for the remaining bats.

	Bat ID													
Tissue	102[1]	108[1]	103	104[2]	109	110[2]	111	112	117	118[2]	123[2]	124[2]	127	128
Brain	None	None	None	None	None	None	None	None	None	None	None	None	None	None
Nares	None	None	None	None	None	None	None	None	None	None	None	None	None	None
Lung	None	None	None	None	None	None	None	None	None	None	None	None	None	None
Heart	None	None	None	None	None	None	None	None	None	None	None	None	None	None
Liver	None	None	None	None	None	None	None	None	None	None	None	None	None	None
Kidney	None	None	None	None	None	None	None	None	None	None	None	None	None	None
Spleen	None	None	None	None	None	None	None	None	None	None	None	None	None	None
Stomach	None	None	None	None	None	None	None	None	None	None	None	None	None	None
Pancreas	None	None	None	None	None	None	None	None	None	None	None	None	None	None
Small intestine	None	None	None	None	None	None	None	None	None	None	None	None	None	None
Colon	None	None	None	None	None	None	None	None	None	None	None	None	None	None

[1]Uninoculated control

[2]SARS-CoV-2 infected bats

None: no detectable SARS-CoV-2 nucleocapsid antigen
